# Synaptic State Matching: A Dynamical Architecture for Predictive Internal Representation and Feature Detection

**DOI:** 10.1371/journal.pone.0072865

**Published:** 2013-08-26

**Authors:** Saeed Tavazoie

**Affiliations:** Department of Biochemistry and Molecular Biophysics, Department of Systems Biology, Columbia University, New York, New York, United States of America; CNRS - Université Claude Bernard Lyon 1, France

## Abstract

Here we explore the possibility that a core function of sensory cortex is the generation of an internal simulation of sensory environment in real-time. A logical elaboration of this idea leads to a dynamical neural architecture that oscillates between two fundamental network states, one driven by external input, and the other by recurrent synaptic drive in the absence of sensory input. Synaptic strength is modified by a proposed synaptic state matching (SSM) process that ensures equivalence of spike statistics between the two network states. Remarkably, SSM, operating locally at individual synapses, generates accurate and stable network-level predictive internal representations, enabling pattern completion and unsupervised feature detection from noisy sensory input. SSM is a biologically plausible substrate for learning and memory because it brings together sequence learning, feature detection, synaptic homeostasis, and network oscillations under a single unifying computational framework.

## Introduction

The search for function of cortical circuits has been a central focus of neuroscience since the pioneering works of Mountcastle [1] and Hubel & Wiesel [2]. A guiding hypothesis behind much of this work is the existence of a canonical microcircuit whose basic computation is critical to sensory processing throughout the cortex. Detailed micro-architectural maps [3] show great promise in advancing our understanding of these circuits. However these efforts will greatly benefit from constraints on the nature of the computational task itself. At the highest level, cognitive systems generate internal representations of the outside world that facilitate adaptive behaviors. To what extent can this high-level truism inform us about neural architecture and computation operating at the very lowest levels? Here I show that a logical extension of this principle, down to the scale of individual synapses, naturally leads to a dynamical neural architecture that generates predictive internal representations, enabling feature detection from noisy sensory input.

To bridge the gap between the abstract notion of internal representations and their physical neural substrate, let us consider *dynamic* internal representations which, in their perfect form, are able to internally generate the recurring spatiotemporal dynamics of sensory input. This internal simulation is limited to those features of the sensory environment that, because of their statistical regularity, are in principle predictable (e.g. moving edges in low-level visual system), Here, sensory input is considered from the perspective of cortex (e.g. activity of Lateral Geniculate Nucleus (LGN) input neurons into V1). Such inputs typically preserve relevant topographic information in two dimensional space (e.g. retinotopy).

How do populations of neurons form a faithful simulation of spatiotemporal dynamics of sensory input? The most obvious substrate is Hebbian synaptic plasticity [4] and its modern variant, spike-timing dependent plasticity (STDP) [5], [6], where a pre-synaptic action-potential (spike) that immediately precedes a post-synaptic spike strengthens the synapse, while one with the opposite temporal order weakens it. In principle, this form of synaptic strength modification would lead to a causal chain of activity reflecting the input pattern. However, learning in model neural networks based on this form of plasticity has clear stability issues since the inherent positive feedback will increase synaptic strength without bounds. Various heuristics have been proposed to address the stability problem by imposing additional constraints on the scale and rate of synaptic potentiation, often utilizing non synapse-local information [5], [7]. The biological plausibility of specific solutions may be in doubt. However, it is clear from experimental evidence that synaptic plasticity strength is somehow homeostatically modulated, presumably in order to maintain neural networks within stable operational bounds [8].

## Results

Arguing from first principles, I will motivate a parsimonious neural architecture design capable of simulating dynamical systems through an inherently stable synaptic modification process operating on strictly local information. Our discrete implementation is composed of a population of neurons with potential all-to-all synaptic connectivity mediated by axons with conduction delay. For the sake of simplicity and symmetry, neurons can form both positive (activating) and negative (inhibitory) synapses onto each other. The neurons are generic McCulloch & Pitts [9] variety whose composite synaptic input determines spiking based on a thresholded sigmoidal response. Sensory input is applied to every neuron in the form of a sequence of spike trains. Recurrent synaptic weights are initially set to zero. The goal is to arrive at a distribution of synaptic weights that enables the network to generate sensory input patterns autonomously. Some form of Hebbian synaptic plasticity could serve as the driving force here. However, we have to ensure that: 1) modifications will lead to robust convergence of synaptic weights to a distribution (*a priori* unknown) that generates accurate predictive internal representations and 2) potentiation will remain within bounds (*a priori* unknown) that ensures long-term network stability.

### Synaptic state matching

Let us focus down on an individual synapse which has access only to local information on pre and post-synaptic spiking events and their temporal correlation-structure over time. From the perspective of this synapse, perfect internal representation of sensory input is achieved when these observable spike statistics are identical between two proposed states: 1) a state in which network activity is generated by sensory input (open), and 2) a state in which network activity is generated by recurrent synaptic connections in the absence of sensory input (closed). I propose that the imposition of this synaptic state-matching (SSM) constraint at individual synapses can give rise to accurate and stable internally generated dynamics at the level of the entire network.

**Figure 1:**
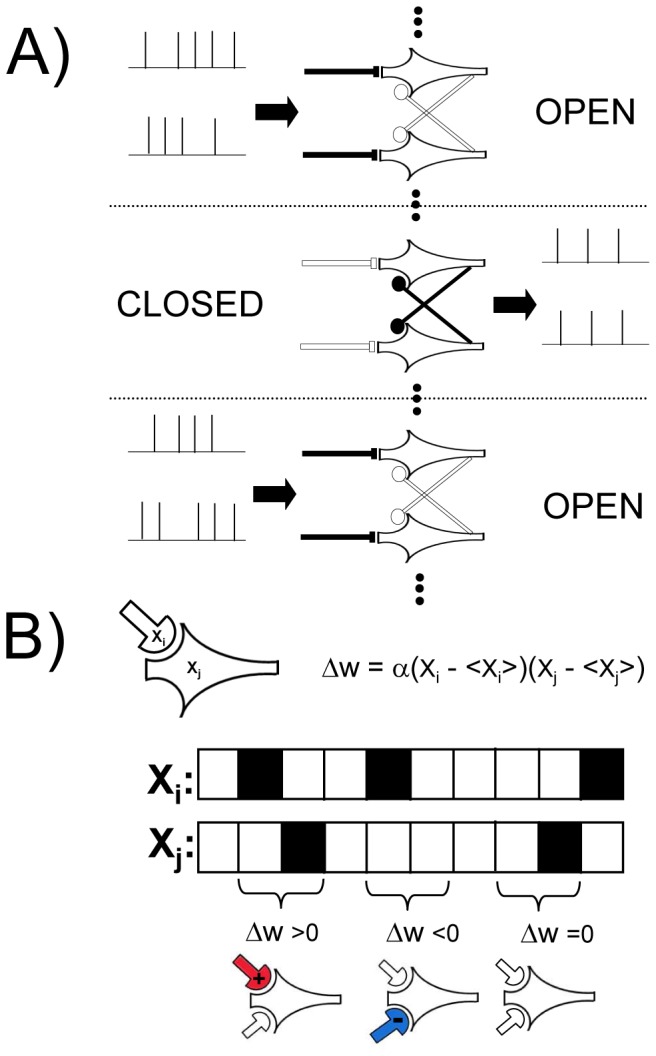
Synaptic state matching architecture and spike-timing dependent covariance plasticity. (*A*) Dynamical architecture of synaptic state switching and state matching. Neurons continually oscillate between two global network states: activity imposed by sensory input (open) and activity generated by recurrent synaptic drive, in the absence of sensory input (closed). (*B*) synaptic plasticity rule (potentiation strength) in discrete time. X_i_ and X_j_ are binary events corresponding to pre-synaptic and post-synaptic spikes, respectively and <X_i_> and <X_j_> are their continuous valued running averages. For an activating synapse (+), a pre-synaptic spike, followed immediately by a post-synaptic spike gives rise to a potential weight increase (Δw>0). In the case of an inhibitory synapse (-), a pre-synaptic spike preceding a quiescent post-synaptic neuron gives rise to a potential weight increase (Δw<0). Potentiation can only occur following a pre-synaptic spike. Candidate potentiation events lead to synaptic strength modulation such that the mean potentiation strength is matched between the open and closed states. As mean potentiation strength is a compact measure of the locally observed spike statistics, synaptic state matching assures that recurrent synaptic drive is recapitulating the spatiotemporal dynamics of sensory input. In this example, spikes correspond to immediate pre and post-synaptic states. In principle, each synapse can have its own axonal conduction delay, in which the presynaptic action potential arrives with a distinct latency relative to the action potential originating at the soma.

To ensure smooth convergence and long-term stability, synaptic state matching occurs in real-time (Figure 1*A*), with the entire network switching back-and-forth between activity imposed by sensory input (*open state*) and that generated by the internal simulation of the most likely sensory input (*closed state*). At each synapse, a presynaptic spike followed closely by a postsynaptic spike will lead to specific biochemical changes with the potential of strengthening the synapse. Let us refer to the magnitude of this effect as ‘potentiation strength’. Let us further assume that each synapse has the capacity to store a running average estimate of its potentiation strength in each of these two distinct states. Such dynamic local storage of information has been previously proposed in the context of reinforcement learning, most notably in Klopf's eligibility trace [10] and Seung's stochastic hedonistic synapse [11].

During the early phase of learning, the closed state is quiescent because weak synapses are unable to generate activity, and potentiation strength computed during this phase is low. During the open state, the strong drive of sensory input generates spiking activity with high potentiation strength among synapses with tightly coupled pre-post spiking. Upon the occurrence of a candidate potentiation event, the synapse makes a decision: the stored mean potentiation strength is compared between the two states; if it is higher in the open state, the synapse is strengthened in proportion to the instantaneous value of potentiation strength; otherwise, if the mean potentiation strength is higher in the closed state, then the synapse has clearly overshot and therefore will correct itself by undergoing a depression penalty, with a scale proportional to its instantaneous potentiation strength. This simple local algorithm ensures that potentiation occurs until pre-post spiking correlations are matched between open and closed states. From the perspective of an individual synapse, this is the best local indication that recurrent synaptic activity is recapitulating the spatiotemporal dynamics of sensory input.

Potentiation strength is computed by a temporally asymmetric Hebbian process that is modulated by the rates of pre- and post-synaptic activity (Figure 1*B*, Figure S1 and Materials and Methods). We call this plasticity rule spike-timing dependent covariance plasticity (STCP) because it is a temporally asymmetric, event-driven, version of covariance learning [12]. In this way, the relative strength of potentiation is proportional to the statistical support for the causal chain of activity from pre-synaptic to post-synaptic neuron. As we show below, this simple local algorithm ensures smooth convergence to synaptic weight distributions that generate accurate and stable dynamic internal representations of sensory input.

### Predictive internal representations

**Figure 2:**
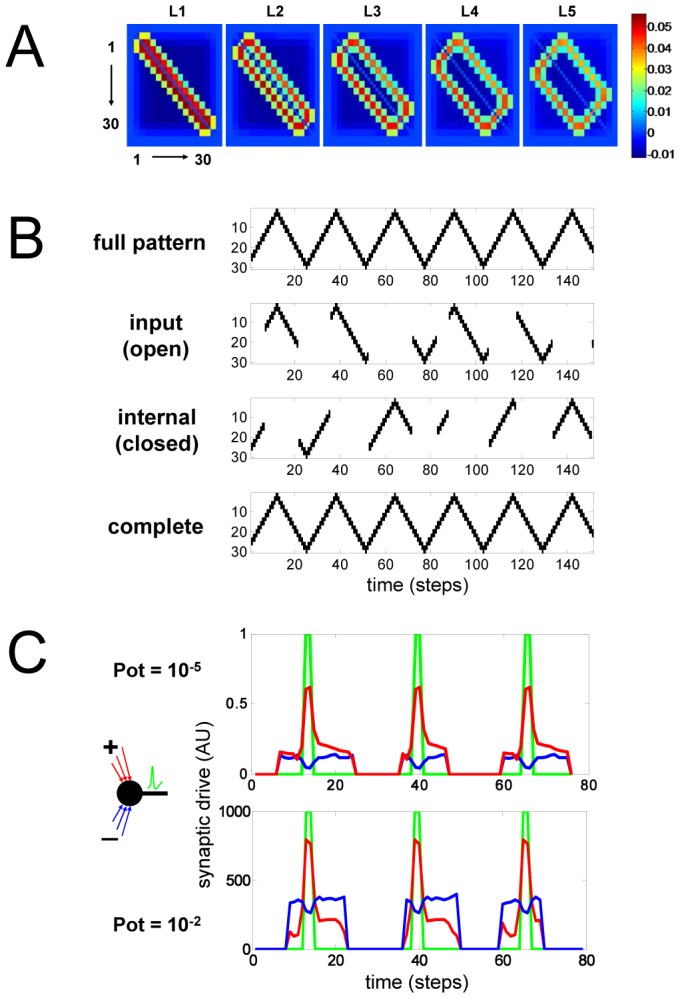
A thirty neuron SSM network trained on input spikes in the form a triangular wave. (*A*) Synaptic weight matrices for different latency conduction delays (1–5 time steps). Positive values (red) correspond to activating connections and negative values (blue) to inhibitory ones. (*B*) full pattern of activity in the sensory environment (full pattern), input activity during the open state (open), internal activity during the closed state (closed), and the combined (complete). Pattern completion during the closed state is a perfect match to the missing input. SSM parameter choices were as follows: potentiation strength (α): 4×10^−5^, spike-rate memory (m_s_): 100, potentiation memory (m_p_): 100, state switching period: (Gaussian, τ_μ_ = 15, τ_σ_ = 5), neuron firing threshold (V_t_): 0.5, sigmoid sharpness (S): 10, latency range (L): 5. Spike-rate memory (m_s_) and potentiation memory (m_p_) are the widths of averaging time window for calculating mean spike-rate and mean potentiation, respectively. (*C*) Input synaptic drive into a single neuron for activating input (red), and inhibitory input (blue). Spikes are represented in green. The comparisons were performed after stabilization of learning. The two simulations are identical except for a thousand-fold higher potentiation scale (α). Each neuron has 290 synaptic inputs (29 neurons ×5 latencies ×2 polarities).

Simulation of a small SSM network demonstrates how predictive internal representations are formed (Figure 2). Details of implementation are available in Materials and Methods. For the sake of demonstration, the input spike trains form a triangular wave pattern with a period of 26 time-steps. Also see Figure S2 for an example of sensory input in the form of repeating circles. The thirty neurons of the network in Figure 2 are driven by sensory input during the open state (Gaussian with mean 15 and s.d. 5 time-steps), and free to generate autonomous activity during the closed state (mean 15 and s.d. 5 time-steps). Over repeated presentations of the stimulus, plasticity drives synaptic weights to a distribution (Figure 2*A*) that leads to generation of internal dynamics. As can be seen, the internally generated pattern is a perfect match to the missing input, in essence a short-term prediction of the most likely (missing) sensory input (Figure 2*B*). In this simulation, every neuron has the potential capacity to interact with every other neuron through connections with time-delay (latencies). The range of latencies (1–5 time-steps) reflects both axonal conduction and synaptic transmission delays that can vary substantially due to axonal diameter/myelination and synapto-dendritic time-constants.

### Robustness to parameter choice, sensory noise, and synaptic perturbations

**Figure 3:**
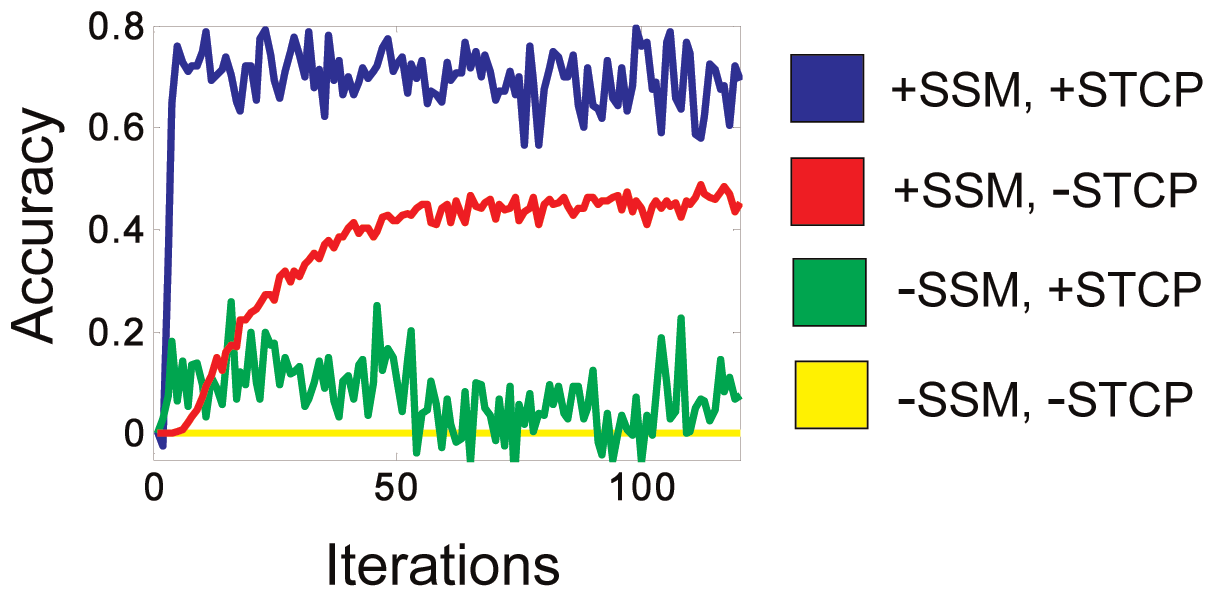
The role of SSM and STCP in the accuracy-trajectory of learning. Synaptic state matching (+SSM) is crucial for accuracy and long term stability. In the (-SSM) simulation, potentiation was unbounded (e.g. unconstrained by comparison of mean potentiation between the open and closed states). Spike-timing dependent covariance plasticity (+STCP) substantially improves convergence rate and accuracy as compared to a plasticity rule that is not modulated by spike-rate history (-STCP). In the (-STCP) simulation, Δw = +/−α. The 40 neuron SSM is trained on two alternating random spike patterns (features), each 40 time-steps long, with an intervening 20 time step quiescent period. Accuracy is a conservative measure of how well internally generated patterns match the missing input during the same time interval. It is defined as one minus the fraction of discordant spikes between the missing input spike trains and the internally generated spike trains. The value here is the average for all forty neurons. Accuracy can be negative in cases where internally generated activity is noisy and/or unstable. The accuracy does not reach maximum (1.0) because the network is unable to generate the earliest part of each random pattern due to the absence of any input during the quiescent period preceding the closed state.

The learning performance is remarkably robust to large variations in almost every parameter, including state-switching frequency, neuron firing threshold, range of latencies, and global potentiation scale (Table S1). For the input pattern in Figure 2, potentiation scale can vary over many orders of magnitude and still give rise to highly accurate and stable dynamics. The key to this remarkable robustness is the synaptic state matching constraint that, although operating locally at individual synapses, ends up imposing absolute constraints on the fidelity of network-level internal representation (Figure 3). One reflection of stability is the near balance of positive and negative synaptic drive into each neuron, across three orders of magnitude in potentiation scale (Figure 2*C*). Experimental evidence suggests that this matching of inhibitory and excitatory drive is a common operating characteristic of neural networks *in vivo* [13], [14].

**Figure 4:**
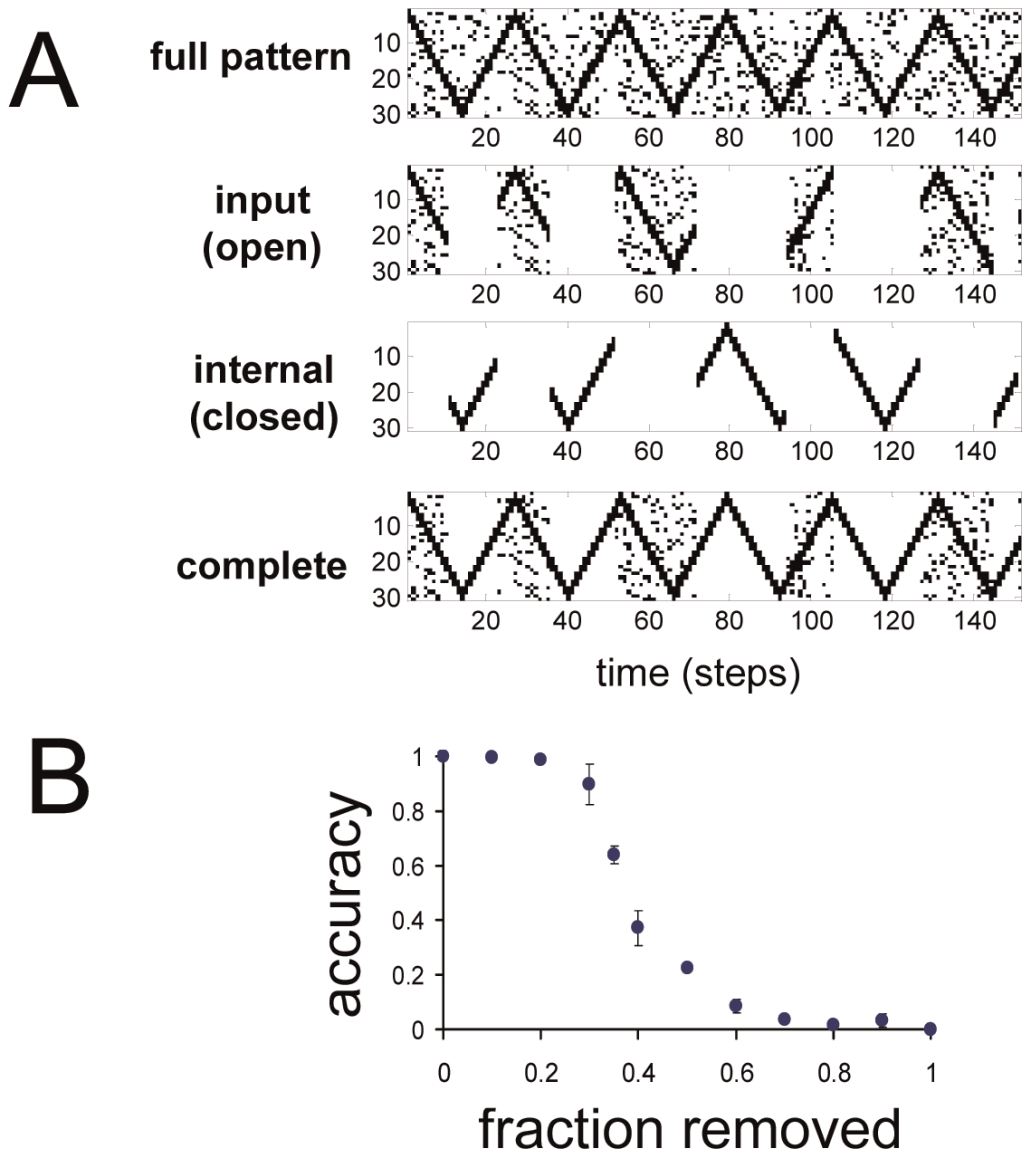
Robustness to sensory noise and synaptic perturbations. (*A*) Pattern of sensory environment with noise (full pattern), input activity during the open state (open), internal activity during the closed state (closed), and the combined (complete). (*B*) Match (accuracy) of internally generated activity to the missing input during the same interval versus fraction of random synapses removed. Accuracy is a conservative measure of how well internally generated patterns match the missing input during the same time interval. It is defined as one minus the fraction of discordant spikes between the missing input spike trains and the internally generated spike trains. The value here is the average for all thirty neurons. Accuracy can be negative in cases where internally generated activity is noisy and/or unstable.

Robustness to input noise and structural perturbations are desirable features of biological neural networks. SSM networks show remarkable noise-resilience, forming accurate and stable internal representations in environments with signal-to-noise ratios (SNR) of ∼1 (Figure 4*A*). As can be seen, this allows SSM networks to perform noise-filtering for stored patterns. It is important to emphasize that, here, by noise we refer to any activity that is not part of a statistically recurring pattern, rather than noise in the traditional sense of the word. Structural perturbations in the form of random synaptic ablation are also well tolerated. In a SSM network trained with the triangular-wave input, thirty percent of synapses can be removed without substantial degradation in performance (Figure 4*B*).

The all-to-all connectivity in the present implementation of SSM networks may seem biologically implausible, especially as networks grow in size. In the relatively small network examples here, the goal was to preserve the *potential* of full connectivity. However, the vast majority of these synapses may not actually be utilized and required for accurate pattern learning. Indeed, the SSM network in Figure 1 is robust to active pruning of the weakest 85% of synapses (Figure S3).

### Detecting multiple spatiotemporal patterns

**Figure 5:**
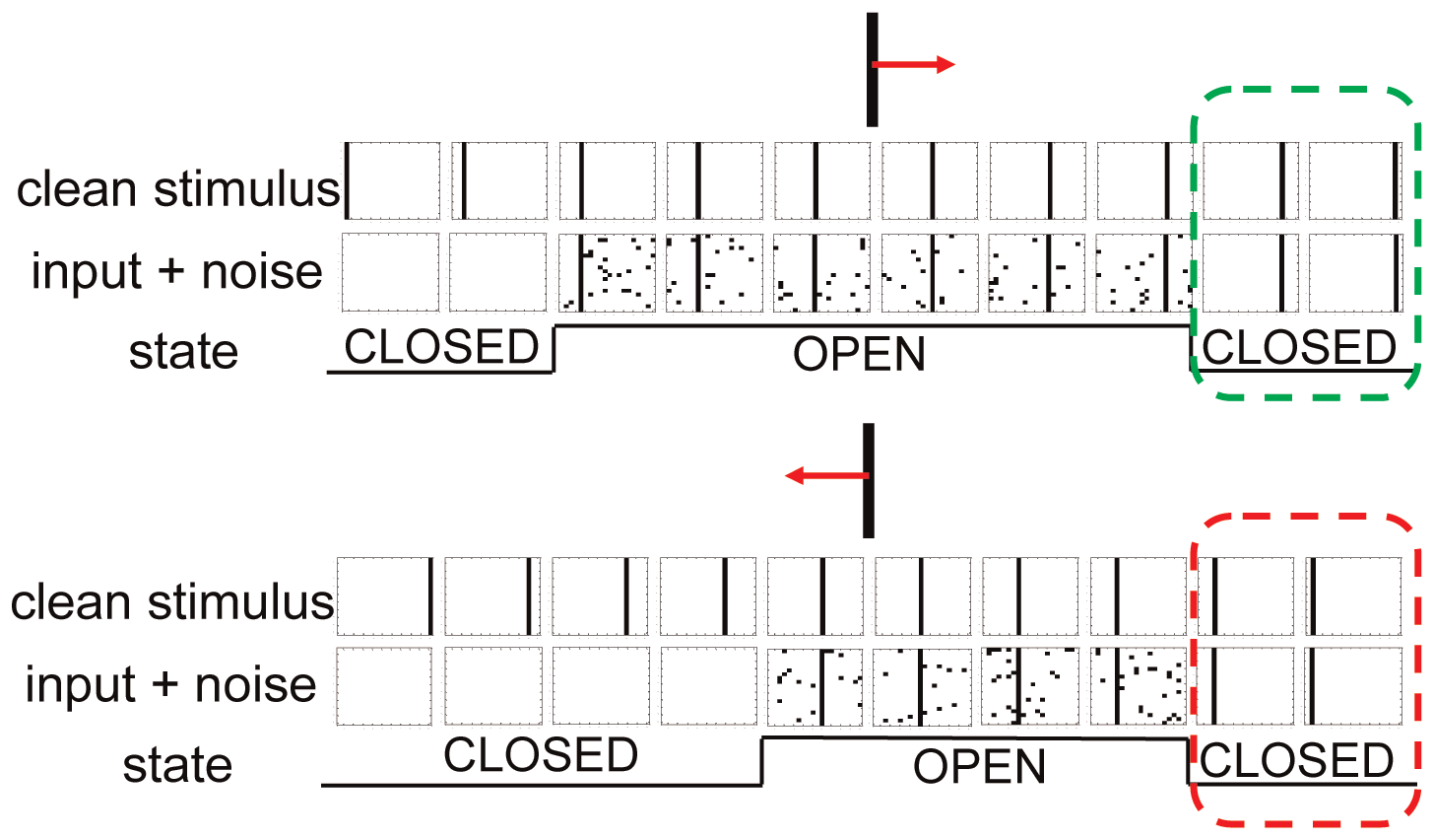
Pattern completion in the context of moving bars across a visual field. A 400 neuron network arranged in a 20×20 sheet with topographic input from the visual field is trained on bars moving left, right, up and down (See Figure S4 for details). The network carries out pattern completion in response to bars moving right (top) and left (bottom). The clean input is presented with equal power random noise during the open state. The network performs perfect pattern completion during the second closed state (dashed boxes) following the presentation of a long-enough sequence of moving bars during the preceding open state. Open/closed durations are sampled from a Gaussian with a mean of 7 and sd of 2 time steps. For the sake of space, bars moving up and down are omitted and only every other time-point is shown.

During the closed state, the network internally generates the most likely input activity pattern, conditioned on the imposed sensory input during the preceding open state. As such, neural activity during the closed state is a short-term prediction of sensory environment. However, the network will not predict the trajectory of sensory environment for all stimuli. Indeed, only recurring patterns with high statistical regularity have the potential of being internalized within the network's synaptic matrix. As such, SSM networks can carry out feature-detection because any activity during the closed state must correspond to pattern completion in response to a *recognizable* feature presented during the preceding open state. In this way, SSM networks filter sensory environment for recurring features on the timescale of state-switching. An illustrative example of this can be seen for spatiotemporal patterns that represent edges (lines) moving in different directions within a simulated visual field (Figure 5*A*, Figure S4). In this example, the neurons are arranged in a 20 by 20 plane, each receiving topographic input from a single retinal photoreceptor. The single 400 neuron SSM network efficiently learns these four patterns and performs robust pattern completion in the presence of noise (Figure 5*A*). In this learning scheme, multiple patterns were presented in an interleaved fashion in order to avoid the, so called, catastrophic interference effect, as previously explored on both theoretical grounds and simulations [15].

### Multi-layer networks

**Figure 6:**
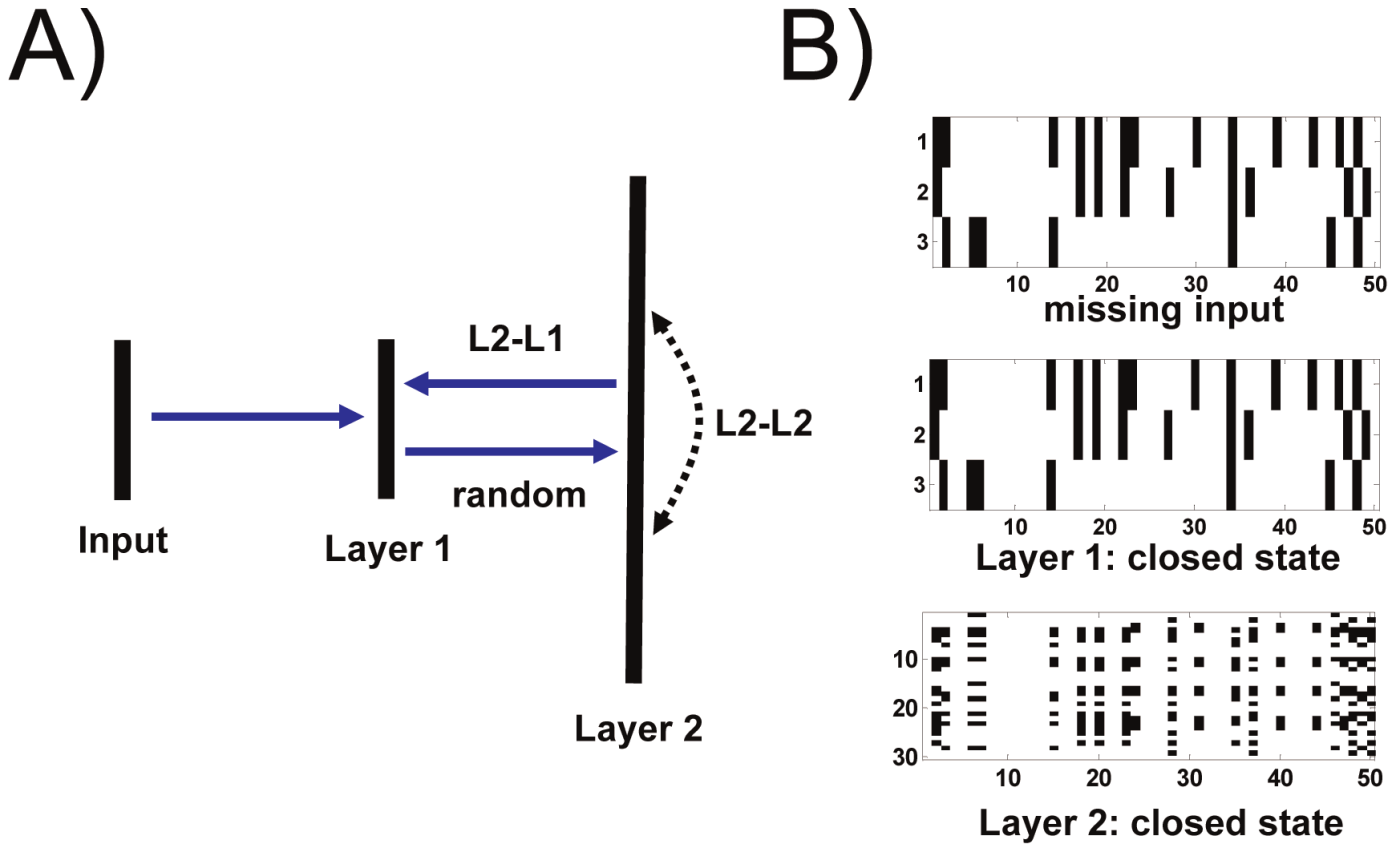
Hidden layer learns a generative model of a long sequence of spike trains. (*A*) The three neurons in layer 1 (Layer 1) are clamped to input spike trains (in this example, a random pattern with spike frequency of 0.2). During the open state, these neurons project (through synaptic weights sampled from a random uniform distribution between -1 and 1) to a much larger hidden layer (Layer 2) which in this example contains thirty neurons. Synaptic state matching in the intra-layer 2 synaptic connections (L2-L2) gives rise to an accurate predictive model within this hidden layer. Synaptic state matching of synapse projections from layer 2 to layer 1 (L2-L1) generates an accurate predictive model of the missing input at layer 1. Layer 1 neurons do not have any internal recurrent connections. (*B*) top: missing input into layer 1 during the closed state; middle: predicted pattern of missing input created by the projection of hidden layer axons to layer 1 during the closed state. The pattern is a perfect match to the missing input; bottom: the coincident activity of the 30 neurons in the hidden layer during the closed state. SSM parameter choices for both L2-L2 and L2-L1 connections were as follows: potentiation strength (α): 5×10^−4^, spike-rate memory (m_s_): 200, potentiation memory (m_p_): 50, state switching period: (Gaussian, τ_μ_ = 30, τ_σ_ = 10), neuron firing threshold (V_t_): 0.5, sigmoid sharpness (S): 10, latency range (L): 20.

**Figure 7:**
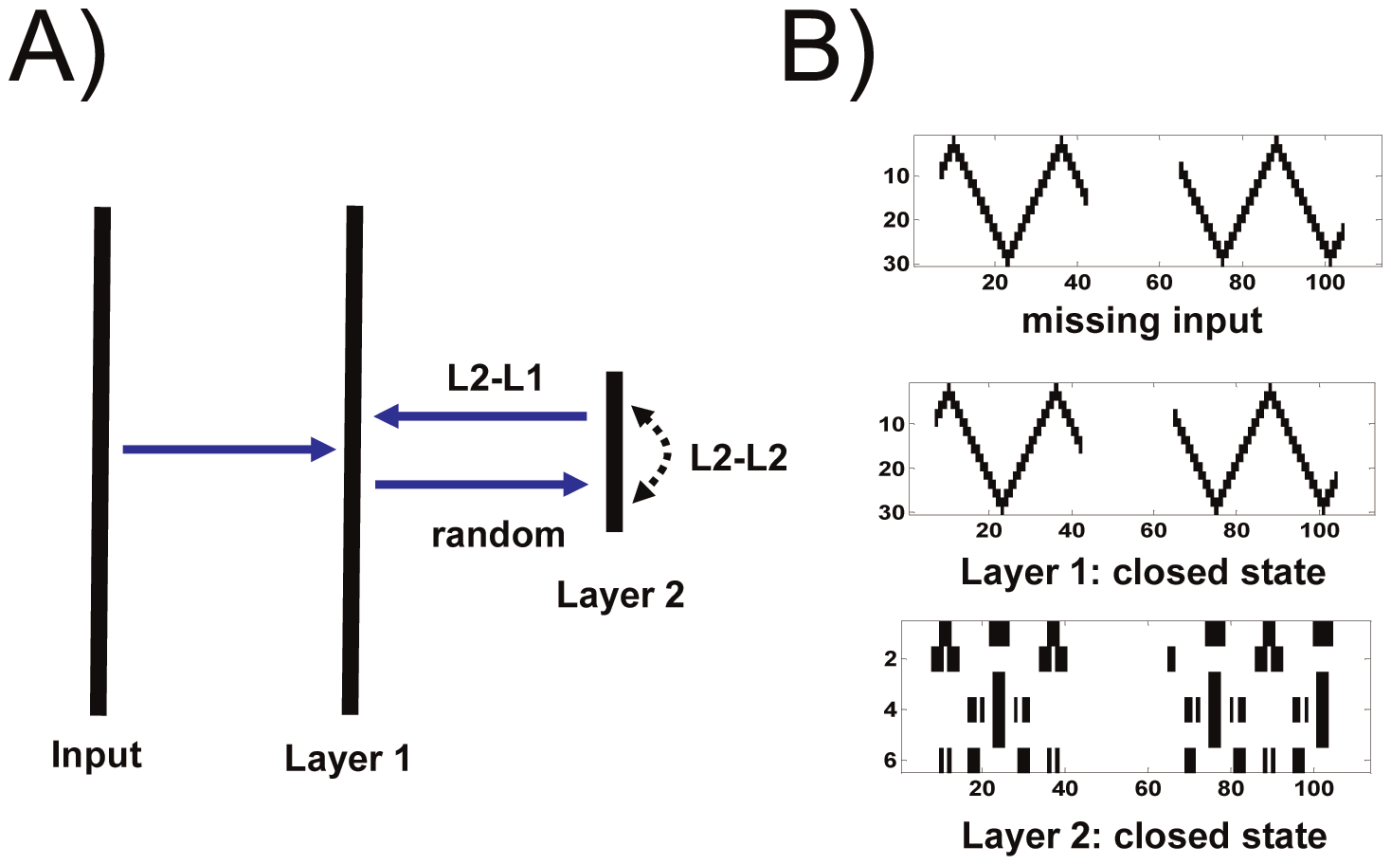
Hidden layer learns a low-dimensional representation of spike trains in the form of a repeating triangular wave. (*A*) The thirty neurons in layer 1 (Layer 1) are clamped to input spike trains in the form a repeating triangular wave. During the open state, these neurons project (through synaptic weights sampled from a random uniform distribution between -1 and 1) to a much smaller hidden layer (Layer 2) which in this example contains six neurons. Synaptic state matching in the intra-layer 2 synaptic connections (L2-L2) gives rise to an accurate predictive model within this hidden layer. In turn, the L2-L1 connections implement a top-down generative model in layer 1. Synaptic state matching of synapse projections from layer 2 to layer 1 (L2-L1) generates an accurate predictive model of the missing input at layer 1. Layer 1 neurons do not have any internal recurrent connections. (*B*) top: missing input into layer 1 during the closed state; middle: predicted pattern of missing input created by the projection of hidden layer axons to layer 1 during the closed state. The pattern is a perfect match to the missing input; bottom: the coincident activity of the six neurons in the hidden layer during the closed state. SSM parameter choices for both L2-L2 and L2-L1 connections were as follows: potentiation strength (α): 5×10^−4^, spike-rate memory (m_s_): 200, potentiation memory (m_p_): 50, state switching period: (Gaussian, τ_μ_ = 30, τ_σ_ = 10), neuron firing threshold (V_t_): 0.5, sigmoid sharpness (S): 10, latency range (L): 20.

The single-layer networks, presented so far, are directly driven by (clamped to) the external input. In this setting, synaptic state matching leads to a generative model that captures correlations in the spatiotemporal dynamics of sensory input. In the realm of machine learning, additional internal hidden layers, with neurons whose activities are not directly clamped to external input, are free to capture higher-order statistical regularities in the distribution of static input patterns [16]. The representational power of SSM networks can be similarly expanded by addition of hidden layers. For example, large hidden layers can capture complexity in a long sequence of spike trains (Figure 6). In turn, small hidden layers can produce lower-dimensional generative models of high-dimensional sensory input (Figure 7).

## Discussion

I propose that a fundamental function of sensory cortex is the generation of an internal simulation of sensory environment in real time. Here, we see that a dynamic neural architecture can achieve this through synaptic state matching driven by a spike-timing dependent covariance plasticity rule. SSM networks accurately internalize the recurring spatiotemporal dynamics of sensory input and thus are able to learn and generate arbitrary temporal sequences. Exposure to sensory input during the open phase has the potential of exciting internally stored sequences. In the absence of sensory input, during the closed state, the network internally generates the most likely short-term prediction of the missing sensory input. We arrived at the dynamical architecture of SSM by first-principle arguments for networks that stably and accurately internalize dynamical systems through synapse-local computations. An interesting outcome of this exercise was that the requisite state-switching is also a convenient substrate for noise-resilient feature detection. Critical to biological plausibility, SSM networks show extreme robustness to parameter choice, input noise, and structural perturbations. In addition, SSM performance scales extremely well to large networks with thousands of neurons and tens of thousands of synapses per neuron.

One can view the learning process as a mapping between a dynamical system (inherent in a recurring pattern within the sensory stream) and the network's synaptic weight matrix. The successful implementation of this mapping by SSM may seem puzzling. How does a step-wise process operating at local synapses end up generating accurate high-dimensional models of dynamical systems? It is helpful to consider the learning process from the perspective of an individual neuron connected within a large population. Any complex recurring pattern of activity in this ensemble, produces time-delayed correlations between the firing of this neuron and all the other neurons in the network. A successful embedding of this pattern within the network requires the *causal* subset of correlations to be somehow translated to synaptic weight strengths that accurately and stably generate the pattern in the absence of input. The temporal asymmetry inherent in spike-timing dependent plasticity (STDP), confines synaptic strengthening to the causal subset of correlations. However, how do we know when to stop strengthening each causal synapse, so as to avoid instability? This is where SSM provides a simple yet powerful solution by locally monitoring a critical measurement: the mean potentiation strength. As soon as a *subset* of causal synapses have been strengthened *enough* such that the mean potentiation strength is *identical* between the open and closed state, the synapse *knows* that some local solution has been achieved. This is because, at this point, pre/post synaptic pair-wise correlations are indistinguishable between the open and closed states. This is the essence of synaptic state matching.

There is an additional consideration. After matching has been achieved, which of the presumably many causally correlated synapses (above noise) should have been significantly strengthened? Here is where the proposed spike-timing dependent covariance plasticity (STCP) provides a principled solution: modulation of potentiation strength by spike-rate history assures that synaptic connections with the highest statistical support for generating the pattern are strengthened *most* (and therefore *first*). Consequently, the learning process is a one-to-many mapping, with each choice of global potentiation scale (α), producing a somewhat different synaptic weight matrix solution for embedding the same pattern. At high values of α, SSM quickly arrives at a solution which means that only the synapses with the strongest correlations are significantly strengthened. At lower values of α, SSM takes longer and the weights are distributed more broadly among many statistically relevant synapses. This flexibility of SSM/STCP is likely a critical contributor to the remarkable parameter robustness seen in our simulations.

### Connections to Machine Learning Algorithms

The utility of bi-phasic network states has been previously exploited in the realm of machine-learning, particularly in the works of Hinton and colleagues on unsupervised learning of generative models [16], [17]. Synaptic state switching has superficial similarities to a phase-switching algorithm used to train Boltzmann machines [16]. As a stochastic extension of Hopfield's associative networks [18], Boltzmann machines have shown great success in modeling the probability distribution of static input patterns. During training, the stochastic units are clamped to temporally uncorrelated input patterns during one phase (learning), and allowed to run freely in the second phase (unlearning). The goal of each phase is to accurately sample pair-wise correlation distributions which, because of the necessity for reaching thermal equilibrium, can take extremely long timescales to accomplish [16]. As such, these training phases have little correspondence to *real-time* state switching and matching in the sense described here. Another machine-learning algorithm with conceptual similarities to SSM is the wake-sleep algorithm used to train Helmholtz machines [17]. Helmholtz machines are used to build a generative model for the distribution of static input patterns in hierarchical neural networks. Bottom-up recognition connections are used to form representations of the input data in one or more hidden layers above. Top-down generative connections are then used to reconstruct approximations to the input using economical hidden representations in the layer(s) above. The learning algorithm switches between the “wake” phase during which a sample from real input data is propagated, first up and then back down the network, and generative weights are modified, and a “sleep” phase during which the network propagates generative samples, first down and then back up the network, and recognition weights are modified. It has been shown that weight modification according to the simple delta rule makes each layer better at reconstructing the activities of the layer below [17].

### Biological implications

Although we present SSM as a generic operating principle throughout the cortex, its fully recurrent architecture and spike-timing internalization best map to the workings of the hippocampus. As such, our study joins a well-established tradition of modeling hippocampal circuits [19]–[21]. Despite its many appealing features, SSM should, nevertheless, be seen as a speculative model of cortical function-a new conceptual framework for interpreting existing observations and generating new hypotheses for experimental exploration. To stimulate these connections it is useful to discuss how the various components of the theory map to biological processes known or postulated to function in real neural networks.

#### Detecting recurring spatiotemporal patterns in the sensory input

Real time state matching is crucial for stability and accuracy (Figure 3), but it also provides a convenient substrate for noise-resilient detection of recurring spatiotemporal patterns in the sensory input. SSM networks split their time between two modes: 1) an observation mode (open state), during which neural activity is influenced by input from sensory environment, and 2) a predictive pattern detection mode (closed state), during which the network generates the most likely sequence of activity, given the preceding observation mode. In this way, each SSM network functions as a *predictive filter* of sensory input because internally generated responses are only elicited to input patterns with high statistical regularity. One can think of these predictable patterns as elementary features of the sensory world, for example moving edges in the visual field. The filtering of complex sensory input for recurring spatiotemporal patterns would be a valuable function in primary sensory cortex because it would focus subsequent processing on the most relevant predictable features of the environment.

#### Oscillations

Do experimental observations support the existence of state-switching? An obvious substrate is network oscillations that span a wide dynamic range in frequency and are ubiquitous in brains [22] including those of insects [23]. One possibility is that the required gating of sensory input is regulated locally by these oscillations. Indeed, there is increasing evidence that the phase of local field oscillations define temporal windows with distinct network connectivity and dynamics [24]. Perhaps the best evidence for such a mechanism is the recently discovered dynamic gating which is coupled to the phase of the local field potentials within the CA1 region of the hippocampus [25]. Alternatively, control could be exerted more centrally, perhaps through the phenomenon of thalamic gating [26].

Cortical oscillations are thought to underlie coordination and synchrony at large spatiotemporal scales. Synaptic state matching would extend the role of oscillations down to the smallest neurobiological scales, at individual synapses and single potentiation events. If the phase of the oscillation defines the open/closed state boundary, SSM would predict that candidate potentiation events are interpreted in a highly phase dependent manner by the synapse. Such phase-dependent plasticity has been experimentally observed in the dentate gyrus of the hippocampus [27]. Furthermore, the matching of potentiation strength by SSM further predicts that candidate potentiation events should induce opposite effects on synaptic strength within each phase. Tantalizing evidence for theta phase-dependent plasticity was observed in the work of Huerta and Lisman using experimental imposition of activity during an LTP/LTD protocol [28]. Bursts of spikes delivered during the peak of the theta oscillation induced LTP, and those delivered during the trough induced LTD [28].

#### Synaptic homeostasis

A major challenge for theoretical models of learning is the lack of a mechanism by which synaptic strength is maintained within operational and stable bounds. An attractive feature of SSM is that synaptic homeostasis is an inherent outcome of state matching. Indeed, real time oscillations between the open and closed state ensure that synaptic strength can be modulated on a rapid timescale in order to produce stable and high-fidelity representation of recurring features in the sensory input. Alternative mechanisms for synaptic homeostasis such as global normalization may produce stable network activity. However, it is unclear how global normalization can preserve accuracy of temporal sequence generation, especially when the same neurons participate in many different cell assemblies (Figure 5).

Real time state matching may not be appropriate at higher levels of the hierarchy because the long periods of disengagement from bottom-up input may have deleterious behavioral consequences. It is tempting to speculate whether the sleep phenomenon may function as the closed state for SSM at longer temporal scales that represent higher-level abstract recurring structures of the sensory environment. This would be a more general version of synaptic downscaling proposed by Tononi and Cirelli [29]. Alternatively, SSM could be implemented during real time awake experience by focal switching, a possible manifestation of which may be the recently discovered local sleep phenomenon in the rat [30]. Perhaps even more speculative is the notion that hallucination states, for example as experienced during psychosis, may be a consequence of defects in the local machinery of synaptic state matching and/or global disturbances in appropriate switching between the open and closed states.

#### Implementation in cortical hardware

Biological realization of SSM requires two essential components: 1) global switching between network states and 2) local synaptic machinery for modulating plasticity. As discussed, cortical oscillations are an appealing source of a network-level signal for switching between open and closed states. In the simplest scenario, state context may be dictated electrically through the phase of the local field potentials. Alternatively, release of chemical modulators that are coupled to these oscillations could signal state context.

During the open state, activity of neurons is strongly influenced by sensory input. During the closed state, sensory input is gated off and neural activity relies solely on recurrent synaptic drive within the network. This switch in operating mode could be dictated locally by the same process that defines state context or, alternatively, by a gating mechanism that functions upstream of the input. Local field oscillations may not, by themselves, allow the gating of external input. However, somatic or synaptic machinery may actively modulate the gain of external inputs based on external LFP phase. In either scenario, there should be evidence for two populations of synapses onto each neuron: 1) a small number of synapses with strong efficacy that receive bottom-up input, and 2) a much larger number of synapses with relatively weak efficacy that are exclusively engaged in recurrent connections. Furthermore, SSM would predict that strongly activating input synapses should exhibit little or no plasticity, relative to the highly plastic recurrent population. These differences in synapse efficacy and potentiation may be defined by a combination of molecular composition and physical location (*e.g.* proximal somatic versus distal dendrites).

SSM also requires synaptic machinery to compute and maintain mean pre and post synaptic activities, along with mean potentiation strength during both open and closed states. Each synapse also requires a molecular decision circuit that determines synaptic modification based on immediate pre/post synaptic activities and a comparison of mean potentiation strength between the open and closed states. Given the complex and highly precise computations performed by biochemical networks, for example as seen in the precise adaptation of bacterial chemotaxis [31], there is little reason to doubt whether synaptic machinery would be capable of carrying out the requisite processes underlying SSM.

## Conclusions

It has been elegantly argued that prediction is an operating principle throughout the brain [32]. Indeed, recent work has shown that even microbial regulatory networks are capable of predictive behavior [33]. Here we see that an atomic prediction principle, locally operating at individual synapses, generates stable and accurate network-level dynamical models that extract recurring features of sensory environments. SSM is an attractive biological mechanism because, in addition to its simplicity, it is remarkably tolerant to noise, structural perturbations, and choice of parameters. Beyond their relevance to cortical information processing, networks based on SSM can serve as general purpose devices in a variety of application domains including sequence learning, sequence generation, feature/object detection, time-series prediction, noise-filtering, and signal decomposition. From the experimental perspective, the search for molecular and cellular correlates of synaptic state matching represents an important area for future research.

## Materials and Methods

### SSM network simulations

SSM networks were simulated in discrete-time using Matlab (Mathworks). Each neuron receives strongly activating synapses from a single input axon. Therefore, during the open state, the activity of each neuron is clamped to the input state and follows it precisely (Figure 1*A*). In an alternative implementation (for example, the hidden layers in Figures 6 and 7), multiple external inputs can converge onto each neuron and the neuron's activity is determined by a weighted sum of all such external inputs during the open state. During the closed state, input is gated off and neuronal activity is determined solely by the incoming synaptic drive from recurrent connections within the network. Here, switching between the two states is determined by a square wave whose open/closed intervals are sampled from a normal distribution with user-defined mean and standard-deviation. Alternatively, the transition between the open and closed state could occur more gradually (e.g. by a sinusoidal function).

In the simplest implementation, every neuron can synapse onto every other neuron through connections with a range of time-delays (e.g. 3–5 time-steps). In biological neural networks, inhibitory synapses are formed by specialized inter-neurons. In our simulations, neurons can directly form both activating and inhibitory synapses. These simpler symmetric networks are essentially equivalent to ones where each excitatory neuron imposes a dominant drive on an inhibitory neuron with spatially extended potential connections in the network.

### Synaptic plasticity

Synaptic weight changes can occur during the open state according to a spike-timing dependent covariance plasticity rule (See details below and Figure S1). Each synapse maintains a local accounting of pre- and post-synaptic spike rates, and of the time-averages of potentiation strength computed under both open and closed states. The ‘aim’ of each synapse is to efficiently drive its synaptic weight to a value that leads to a close matching of the mean potentiation strength between the open and closed states. This is each synapse's best attempt at producing an internal representation given that it only has access to local information. Parameters spike-rate memory and potentiation memory determine the time-scale of the running average for spike-rate and potentiation strength, respectively.

If the scale of the step-wise change in synaptic strength (determined by α) is large, the synapse may overshoot its optimal weight. This may be reflected in a mean potentiation strength that is higher in the closed state as compared to the open state. In such a scenario, the synapse will undergo a weight depression penalty proportional to its instantaneous potentiation strength. This local homeostatic mechanism ensures long-term synaptic state matching which, in turn, guarantees long-term accuracy and stability (Figure 3).

### Details of implementation

#### Neuron model

For each neuron, contributions from all synapses are added linearly, proportional to their weights, and a sigmoidal function with a threshold determines spiking: 
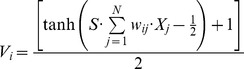


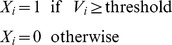



Here, *V_i_* is the membrane voltage of neuron *i*. *w_ij_* is the synaptic weight (strength) of connection from neuron *j* to neuron *i*. *X_j_* is the spiking state of neuron *j*. *N* is the total number of neurons and parameter *S* determines the sharpness of the hyperbolic tangent sigmoid function. If the membrane voltage reaches threshold, the neuron fires.

#### Learning

Learning happens locally at the level of each synapse. Each synapse has the capacity to compute a ‘potentiation strength’ which depends on immediate pre-synaptic and post-synaptic states and their averages over time, according to: P_ij_ = (X_i_ - <X_i_>). (X_j_ - <X_j_>). P_ij_ is the potentiation strength between a pre-synaptic neuron j and a post-synaptic neuron i. X_i_ is the state of the post-synaptic neuron. X_j_ is the past state of the pre-synaptic neuron at a time-delay determined by the synapse's latency. <X_i_> is the time-average of X_i_. <X_j_> is the time-average of X_j_. In our discrete-time implementation, at time point (t), potentiation is computed for value of X_i_ at time point (t) and the immediately preceding value of X_j_ at time point (t-1). In any continuous-time implementation, a different temporally asymmetric rule can apply, for example classical STDP [6].

Each synapse has the capacity to store a running average of its potentiation strength in the two different global network states (open and closed). These “mean potentiation strength” values are utilized in order to carry out synaptic state matching (SSM).

#### Synaptic state matching

SSM is achieved by modulating the strength of each synapse (w_ij_) according the following rule: 
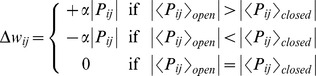



#### Condition 1

if the mean potentiation strength is higher in the open state, relative to its value in the closed state, then the weight of the synapse is increased in proportion to its instantaneous value of potentiation strength. A global parameter called potentiation-scale (α) determines this proportionality constant.

#### Condition 2

if the mean potentiation strength is higher in the closed state, relative to its value in the open state, then the weight of the synapse is decreased in proportion (α) to its instantaneous value of potentiation strength.

#### Condition 3

if mean potentiation strength is equal between the open and closed states, the weight of the synapse is not modified.

For each latency, there are two types of synapses, activating (positive) and inhibitory (negative). A positive value of potentiation strength has the potential of strengthening the weight of an activating synapse. A negative value of potentiation strength has the potential of strengthening the weight of an inhibitory synapse.

Over time, the learning process will culminate in synapse weights that ensure matching of mean potentiation strength between the open and closed states. This, in turn, gives rise to a matching of the statistics of neuron state dynamics between the open and closes states, and, in turn, the ability of the network to generate predictive internal representations of external input during the closed state.

#### SSM network simulator

A Matlab (Mathworks) implementation with graphical user interface allows simulation of SSM networks with hundreds of neurons on a typical CPU.

## Supporting Information

Figure S1 **Spike-timing dependent covariance plasticity (STCP).** Following every presynaptic event (X = 1), potentiation strength is computed by a temporally asymmetric event-driven Hebbian process that takes into account the average activities of presynaptic (<Xi>) and postsynaptic (<Xj>) neurons. In this cartoon example, three potential scenarios are depicted: (1) a presynaptic spike, immediately followed by a post-synaptic spike, gives rise to a positive potentiation strength (Δw>0) with the potential of strengthening an activating synapse. (2) a presynaptic spike immediately followed by a quiescent postsynaptic neuron, gives rise to a negative potentiation strength (Δw<0) with the potential of strengthening an inhibitory synapse. (3) In the absence of a presynaptic event, potentiation strength is zero (Δw = 0).
(TIF)

Figure S2 **A 30 neuron SSM network trained on input spikes in the form of repeating circles.** (*A*) Input presented to the network during the open state (open). The network generates internal activity during the closed state (closed). This SSM network was trained with the potential of long latency synaptic connections (1–20). (*B*) Synaptic weight matrices for conduction-delay (latencies) of 1–20 time steps. SSM network parameter choices were as follows: potentiation strength (α): 5×10^−5^, spike-rate memory (ms): 50, potentiation memory (mp): 50, state switching period: (Gaussian, τ_μ_ = 15, τ_σ_ = 5), neuron firing threshold (Vt): 0.5, sigmoid sharpness (S): 10, latency range (L): 20.
(TIF)

Figure S3 **Learning accuracy as a fraction of active pruning of a fraction of synapses.** The weights of the weakest fraction of synapses in the SSM network presented in Figure 2 are actively pruned (set to zero) and accuracy determined. The network preserves accurate pattern learning while the weakest 85% of its synapses are continually set to zero during the learning process.
(TIF)

Figure S4 **An SSM network for moving bars across a model visual field.** Top left: sensory input into a two-dimensional field (20×20) generates activities in 400 neurons arranged as shown. Right: synaptic weight matrices (for latencies 1–15). Each of these is a 400 by 400 matrix of synaptic weights (bottom left for latency  = 1). Positive values correspond to activating synapses, and negative values to inhibitory synapses. SSM parameter choices were as follows: potentiation strength (α): 5×10^−5^, spike-rate memory (ms): 50, potentiation memory (mp): 50, state switching period: (Gaussian, τ_μ_ = 7, τ_σ_ = 2), neuron firing threshold (Vt): 0.5, sigmoid sharpness (S): 10, latency range (L): 15. Each neuron has 11970 synaptic inputs (399 neurons ×15 latencies ×2 polarities).
(TIF)

Table S1 **Parameter insensitivity.** Operational ranges that maintain accuracy above 0.80 on the single triangular wave input pattern (Fig. 2). Spike-rate memory and potentiation memory are the widths of averaging time window for calculating mean spike-rate and mean potentiation, respectively. Asterisks denote conservative bounds because values beyond were not tested.
(DOC)
