# Supplementary figures and images for: Synaptic State Matching: A Dynamical Architecture for Predictive Internal Representation and Feature Detection
Source: PLoS One. 2013 Aug 26;8(8):e72865. doi: 10.1371/journal.pone.0072865 (PMC3753233; doi:10.1371/journal.pone.0072865)

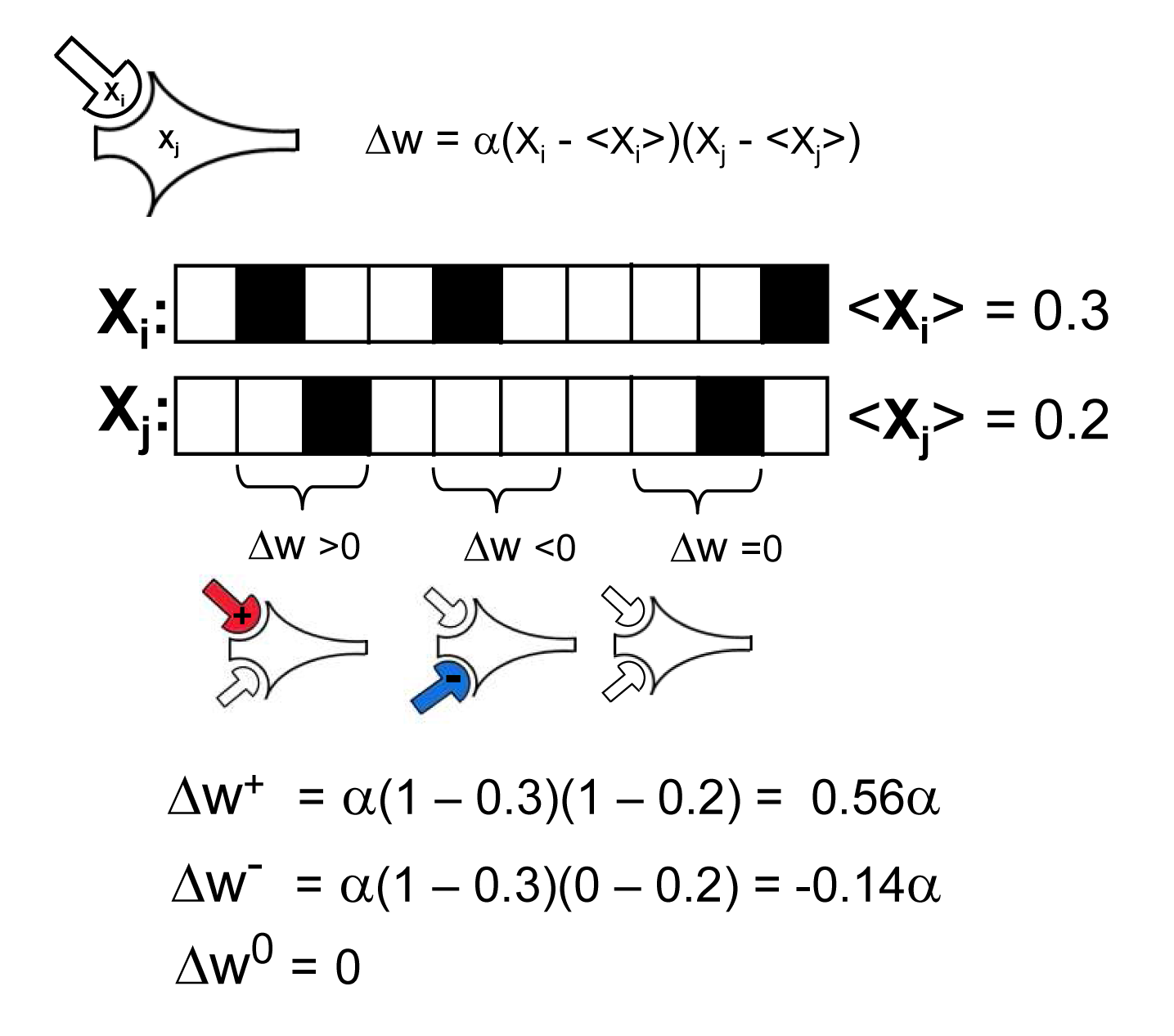

Supplement: Figure S1 — Spike-timing dependent covariance plasticity (STCP). Following every presynaptic event (X = 1), potentiation strength is computed by a temporally asymmetric event-driven Hebbian process that takes into account the average activities of presynaptic () and postsynaptic () neurons. In this cartoon example, three potential scenarios are depicted: (1) a presynaptic spike, immediately followed by a post-synaptic spike, gives rise to a positive potentiation strength (Δw>0) with the potential of strengthening an activating synapse. (2) a presynaptic spike immediately followed by a quiescent postsynaptic neuron, gives rise to a negative potentiation strength (Δw<0) with the potential of strengthening an inhibitory synapse. (3) In the absence of a presynaptic event, potentiation strength is zero (Δw = 0). (TIF) [file pone.0072865.s001.tif]

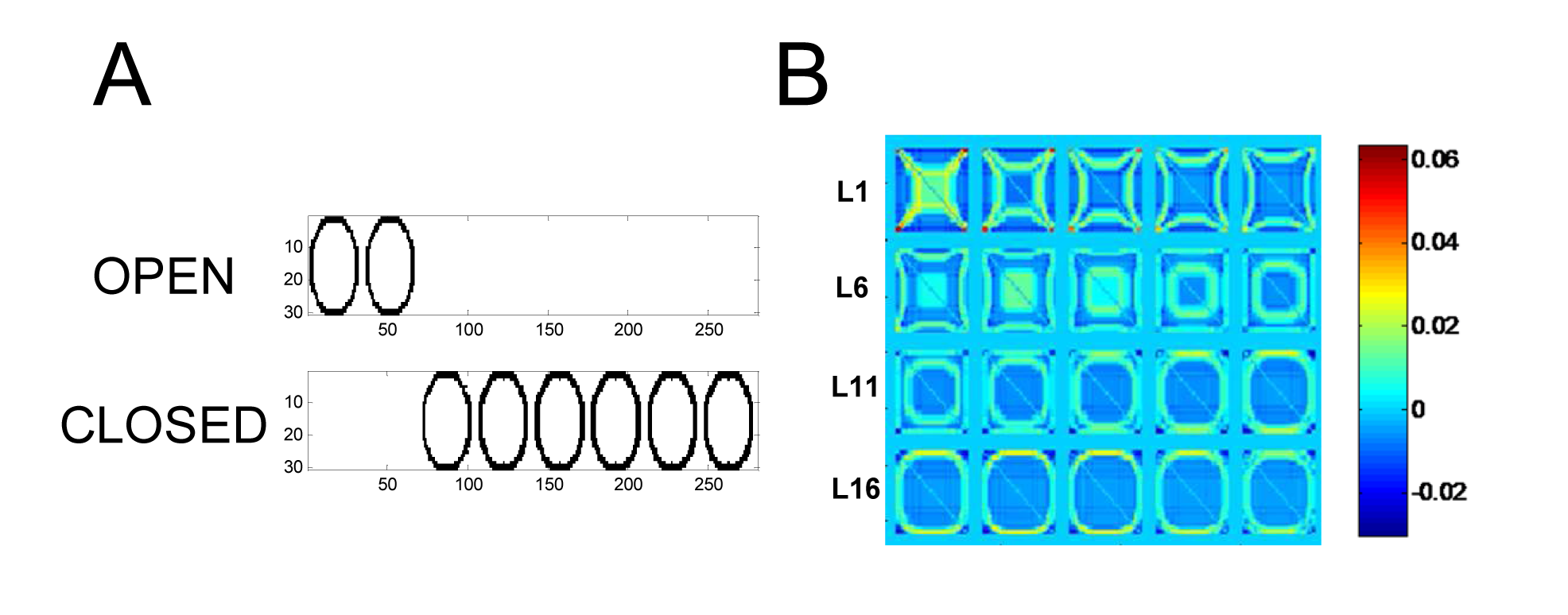

Supplement: Figure S2 — A 30 neuron SSM network trained on input spikes in the form of repeating circles. (A) Input presented to the network during the open state (open). The network generates internal activity during the closed state (closed). This SSM network was trained with the potential of long latency synaptic connections (1–20). (B) Synaptic weight matrices for conduction-delay (latencies) of 1–20 time steps. SSM network parameter choices were as follows: potentiation strength (α): 5×10^−5^, spike-rate memory (ms): 50, potentiation memory (mp): 50, state switching period: (Gaussian, τμ = 15, τσ = 5), neuron firing threshold (Vt): 0.5, sigmoid sharpness (S): 10, latency range (L): 20. (TIF) [file pone.0072865.s002.tif]

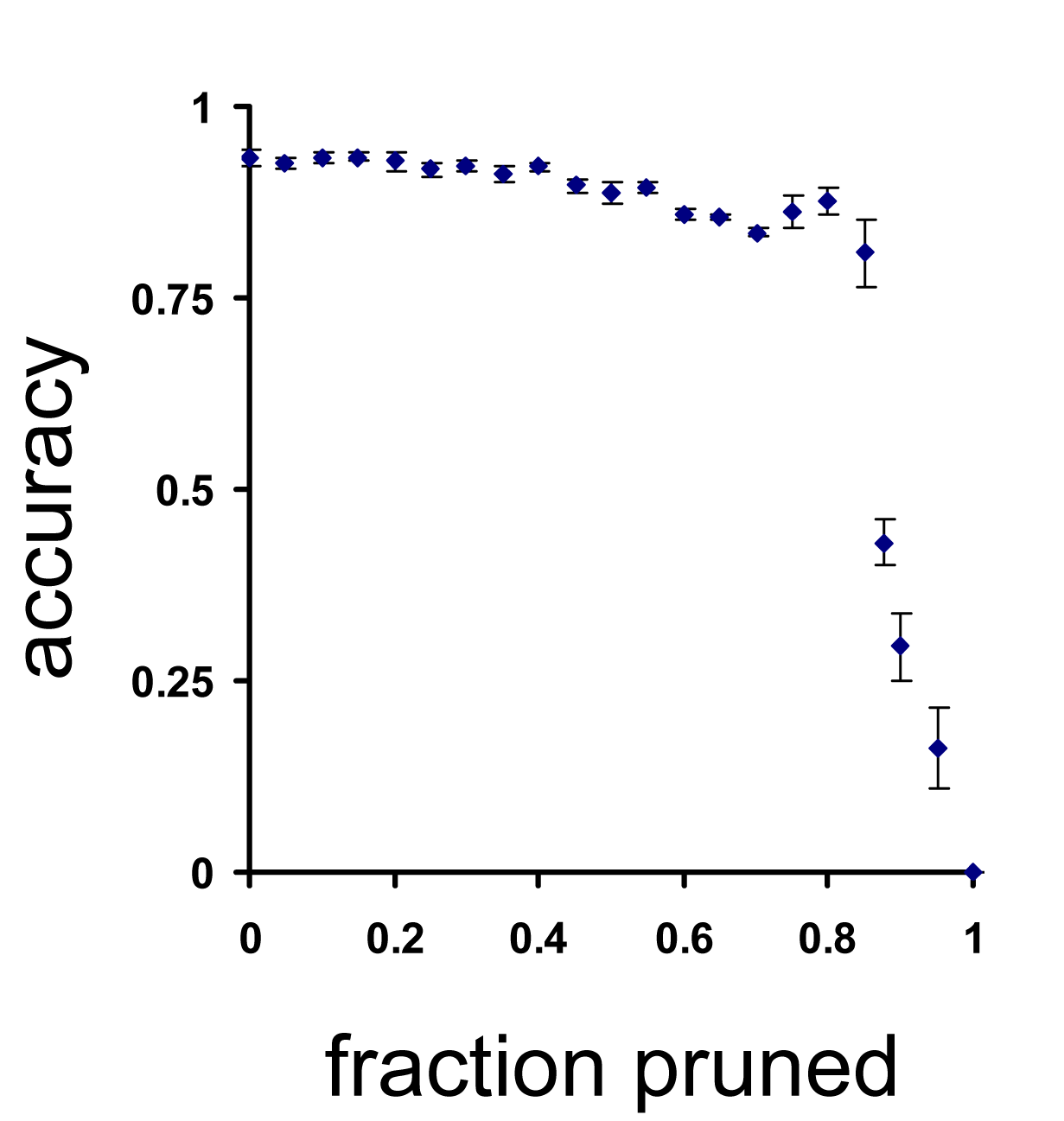

Supplement: Figure S3 — Learning accuracy as a fraction of active pruning of a fraction of synapses. The weights of the weakest fraction of synapses in the SSM network presented in Figure 2 are actively pruned (set to zero) and accuracy determined. The network preserves accurate pattern learning while the weakest 85% of its synapses are continually set to zero during the learning process. (TIF) [file pone.0072865.s003.tif]

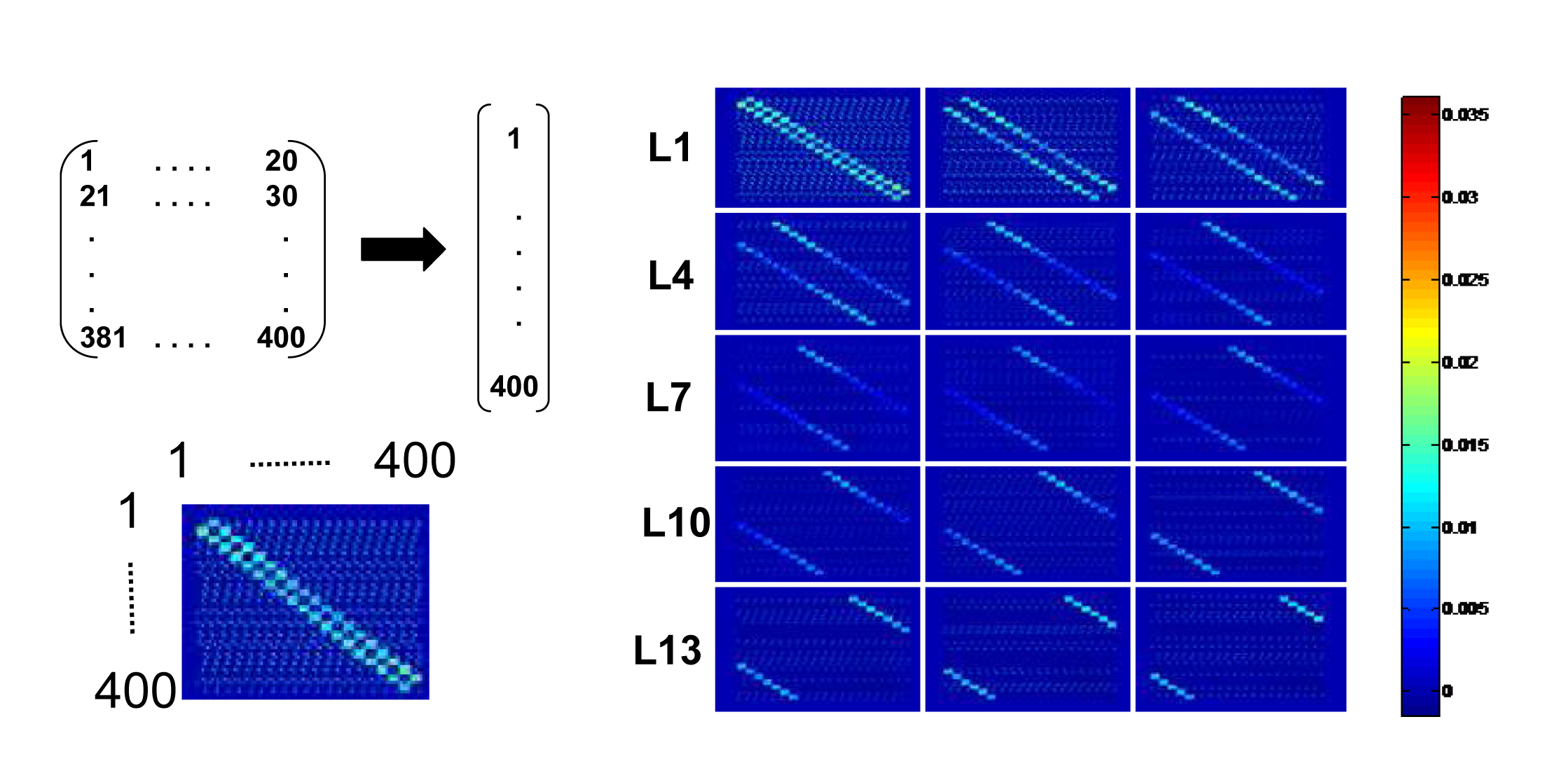

Supplement: Figure S4 — An SSM network for moving bars across a model visual field. Top left: sensory input into a two-dimensional field (20×20) generates activities in 400 neurons arranged as shown. Right: synaptic weight matrices (for latencies 1–15). Each of these is a 400 by 400 matrix of synaptic weights (bottom left for latency = 1). Positive values correspond to activating synapses, and negative values to inhibitory synapses. SSM parameter choices were as follows: potentiation strength (α): 5×10^−5^, spike-rate memory (ms): 50, potentiation memory (mp): 50, state switching period: (Gaussian, τμ = 7, τσ = 2), neuron firing threshold (Vt): 0.5, sigmoid sharpness (S): 10, latency range (L): 15. Each neuron has 11970 synaptic inputs (399 neurons ×15 latencies ×2 polarities). (TIF) [file pone.0072865.s004.tif]
