# Supplementary material for: Synaptic State Matching: A Dynamical Architecture for Predictive Internal Representation and Feature Detection
Source: PLoS One. 2013 Aug 26;8(8):e72865. doi: 10.1371/journal.pone.0072865 (PMC3753233; doi:10.1371/journal.pone.0072865)
Supplement: Table S1 — Parameter insensitivity. Operational ranges that maintain accuracy above 0.80 on the single triangular wave input pattern (Fig. 2). Spike-rate memory and potentiation memory are the widths of averaging time window for calculating mean spike-rate and mean potentiation, respectively. Asterisks denote conservative bounds because values beyond were not tested. (DOC) [file pone.0072865.s005.doc]

| **Parameter** | **Operational Range** |
| --- | --- |
| mean state switching period (τ_μ_) | 7 - 240^*^ |
| neuron firing threshold (V_t_) | 0.1 - 0.7 |
| sigmoid sharpness (S) | 1 - 100^*^ |
| latency (L) | > 3 |
| potentiation scale (α) | 5x10^-5^ - 10^*^ |
| spike-rate memory (m_s_) | 5-5000^*^ |
| potentiation memory (m_p_) | 5-5000^*^ |
